# A Novel Proteogenomic Integration Strategy Expands the Breadth of Neo-Epitope Sources

**DOI:** 10.3390/cancers14123016

**Published:** 2022-06-19

**Authors:** Haitao Xiang, Le Zhang, Fanyu Bu, Xiangyu Guan, Lei Chen, Haibo Zhang, Yuntong Zhao, Huanyi Chen, Weicong Zhang, Yijian Li, Leo Jingyu Lee, Zhanlong Mei, Yuan Rao, Ying Gu, Yong Hou, Feng Mu, Xuan Dong

**Affiliations:** 1 College of Life Sciences, University of Chinese Academy of Sciences, Beijing 100049, China; xianghaitao@genomics.cn (H.X.); guanxiangyu@genomics.cn (X.G.); zhangweicong@genomics.cn (W.Z.); liyijian@genomics.cn (Y.L.); 2 BGI-Shenzhen, Shenzhen 518103, China; bufanyu@genomics.cn (F.B.); aloha.chenlei@gmail.com (L.C.); zhanghaibo3@genomics.cn (H.Z.); zhaoyuntong@genomics.cn (Y.Z.); chenhuanyi@genomics.cn (H.C.); guying@genomics.cn (Y.G.); 3 BGI-GenoImmune, BGI-Shenzhen, Shenzhen 518083, China; zhangle@genomics.cn (L.Z.); leeleojingyu@genomics.cn (L.J.L.); 4 Guangdong Provincial Key Laboratory of Human Disease Genomics, Shenzhen Key Laboratory of Genomics, Shenzhen 518083, China; 5 BGI, Shenzhen 518083, China; meizhanlong@genomics.cn (Z.M.); raoyuan@genomics.cn (Y.R.); houyong@mgi-tech.com (Y.H.); 6 Guangdong Provincial Key Laboratory of Genome Read and Write, Shenzhen 518120, China

**Keywords:** mass spectrometry, immunopeptidome, neo-epitope, immunotherapy

## Abstract

**Simple Summary:**

Tumor-specific antigens are ideal targets for cancer immunotherapy. Mass spectrometry, which is the main method that directly identifies neo-epitopes presented on tumor cells, focuses mainly on peptides derived from annotated protein-coding exomes. However, non-canonical peptides arising from alterations at genomic, transcriptional, and posttranslational levels have been identified in several pioneering studies, making it necessary to develop an integrated proteogenomic approach that can comprehensively identify neoantigens derived from all genomic regions. Our novel strategy combining database searches with a de novo peptide sequencing method accurately identified multiple types of non-canonical peptides in the colorectal cancer cell line, HCT116. This practical proteogenomic strategy can be applied to neoantigen discovery in clinical tumor samples, improving cancer immunotherapy.

**Abstract:**

Tumor-specific antigens can activate T cell-based antitumor immune responses and are ideal targets for cancer immunotherapy. However, their identification is still challenging. Although mass spectrometry can directly identify human leukocyte antigen (HLA) binding peptides in tumor cells, it focuses on tumor-specific antigens derived from annotated protein-coding regions constituting only 1.5% of the genome. We developed a novel proteogenomic integration strategy to expand the breadth of tumor-specific epitopes derived from all genomic regions. Using the colorectal cancer cell line HCT116 as a model, we accurately identified 10,737 HLA-presented peptides, 1293 of which were non-canonical peptides that traditional database searches could not identify. Moreover, we found eight tumor neo-epitopes derived from somatic mutations, four of which were not previously reported. Our findings suggest that this new proteogenomic approach holds great promise for increasing the number of tumor-specific antigen candidates, potentially enlarging the tumor target pool and improving cancer immunotherapy.

## 1. Introduction

Neoantigens are promising immunotherapy targets. They are specifically presented on tumor cells by human leukocyte antigens (HLAs), also known as major histocompatibility complexes (MHC), and are considered safe and potent targets for T cell-based immunotherapies [1,2,3]. Neoantigens can be classified into two main types based on the variant sources: canonical antigens encoded within the open reading frames (ORFs) of protein-coding genes [4]; and non-canonical antigens (also called alternative, cryptic, or dark-matter antigens) derived from various alterations at the genomic, transcriptomic, or proteomic level [5,6].

The most common strategies for identifying tumor-specific antigens (TSAs) have generally relied on in silico neoantigen prediction and functional screening to verify the presentation and immunogenicity of the neoantigens. Neoantigen prediction is based solely on computational tools that predict the binding affinity between peptides and HLA allotypes by performing genome variant analysis using the whole-exome sequencing (WES) data [7,8,9]. This approach focuses on peptides derived from somatic mutations, missing non-canonical epitope candidates. Although this results in a large number of predicted peptide ligands, only approximately 6% at most have been shown to be immunogenic [9,10]. Additionally, this technique often yields false-positive neo-epitopes, almost 90% of which are not present on the cell surface [11,12]. 

Liquid chromatography with tandem mass spectrometry (LC-MS/MS) is currently the main method that can be used to directly identify neoantigens that are naturally processed and presented by tumor cells. The peptide-MHC complexes are co-immunoprecipitated with HLA I and/or HLA II antibodies, and the peptides are eluted and analyzed using mass spectrometry. The LC-MS/MS-based method combining conventional proteomics with genomic data for neoantigen identification (referred to as proteogenomics [13]) has been used in the analysis of several tumors, including melanoma [14], colorectal cancer [15], and neuroblastoma [16], but only a few TSAs were shown to be immunogenic. In melanoma, for example, eleven mutated peptides were identified in five patients, and only four were immunogenic [14]. In a colorectal cancer (CRC) study, only one mutated peptide was shown to be immunogenic in one mismatch repair-deficient CRC patient, and none was found in the other five mismatch repair-proficient CRC patients [15]. Although combining LC-MS/MS with genomics strategies can compensate for the low accuracy of in silico neo-epitope identification, it often only focuses on identifying “neoantigens” generated by somatic mutations in annotated coding genes alone, whereas tumor neoantigens can also originate from alterations occurring at genomic [17,18], transcriptomic [19] or proteomic [20] levels. This limitation in neoantigen identification has spawned the search for various sources of neoantigens arising from dysregulated translation events [21], which requires the incorporation of a variety of mutational information into the database prior to mass spectrometry data analysis. However, simply adding mutation information to the database without any filtering greatly inflates the search space, which in turn leads to an underestimation of the true FDR of the variant peptide [22]. On the other hand, most proteomic approaches do not combine the irreplaceable advantages of de novo peptide sequencing to discover neo-epitopes [15,23,24]. It is therefore necessary to develop an integrated approach that can identify neoantigens from all possible origins. 

Here, we present a novel proteogenomic integration strategy that combines multiple database search engines with a de novo peptide sequencing method. The individualized database for database search incorporates somatic mutation from all genome regions to eliminate peptides that are unlikely to be presented by HLA, avoiding an inflated search space and improving the sensitivity and accuracy of the identification. Our strategy can identify various non-canonical peptides, including spliced peptides and linear peptides derived from intergenic regions, intronic regions, etc., which greatly increases the number of peptides that can be identified by LC-MS/MS techniques. 

We implemented this strategy to study 11 batches of HCT116-derived mass spectrometry (MS) data and achieved robust performance outcomes in the identification of canonical epitopes as well as high confidence noncanonical epitopes. The predicted retention times of the identified peptides are highly consistent with the observed retention times, with a Pearson correlation coefficient of up to 0.969. All of the 8 identified mutation-bearing peptides were confirmed by synthetic validation experiment, 6 of which were further detected by parallel reaction monitoring (PRM) mode. This novel integrated strategy expands the breadth of potential neo-epitopes and can accelerate the discovery of targets for cancer vaccines and T cell-based immunotherapy.

## 2. Materials and Methods

### 2.1. Cell Lines

The HCT116 cell line was purchased from the American Type Culture Collection and maintained in DMEM medium (Gibco, Grand Island, NY, USA) with 10% fetal bovine serum (ExCell Bio, Clearwater, FL, USA) and 1% penicillin-streptomycin solution (Gibco) in a 5% CO_2_ atmosphere at 37 °C. Cells were grown to the required cell volume (1–5 × 10^8^), harvested, centrifuged at 1000× *g* for 10 min, washed twice with ice-cold PBS, and stored as dry cell pellets at −80 °C until processing.

### 2.2. Immunoprecipitation of HLA-I Complexes

HLA-I complexes were harvested as previously described [25]. In brief, the cell pellets were lysed at 4 °C for 1 h with phosphate-buffered saline (Gibco) containing 0.5% sodium deoxycholate (Sigma-Aldrich, St. Louis, MO, USA), 2% octyl-β-D-glucopyranoside (Sigma-Aldrich), protease inhibitor cocktail (Roche, Basel, Switzerland), 2 mM PMSF (Beyotime, Jiangsu, China), 2 mM EDTA (Invitrogen, Carlsbad, CA, USA), and 0.4 mM iodoacetamide (Sigma-Aldrich). The lysates were centrifuged at 17,000× *g* and 4 °C for 50 min. HLA class I complexes were immunoaffinity-purified from the supernatant using the W6/32 antibody (AtaGenix, Wuhan, China) cross-linked to protein A Sepharose CL-4B beads (Cytiva, Marlborough, MA, USA) in a 96-well filter microplate (Agilent, Santa Clara, CA, USA) preconditioned with 100% acetonitrile (CAN), 0.1% trifluoroacetic acid (TFA), and 100 mM Tris-HCl, pH 8.0. The beads were then subjected to a series of washes at room temperature (about 23 °C) as follows: 4 washes using 2 mL buffer A (150 mM NaCl, 20 mM Tris-HCl pH 8.0), 4 washes using 2 mL buffer B (400 mM NaCl, 20 mM Tris-HCl pH 8.0), 4 washes using 2 mL buffer A, and 2 washes using 2 mL buffer C (20 mM Tris-HCl pH 8.0). HLA class I complexes and bound peptides were eluted with 1% TFA. The eluate was loaded onto Waters Sep-Pak tC1896-well cartridges (Waters, Milford, MA, USA) that had been pre-washed and equilibrated with 80% ACN in 0.1% TFA and then washed twice with 0.1% TFA. Before purifying the bound peptides, the cartridges were washed first with 0.1% TFA, and then with 2% ACN in 0.1% TFA. The peptides were eluted from the cartridges using 14% ACN in 0.1% TFA and 28% ACN in 0.1% TFA. The collected peptides were dried using centrifugal vacuum concentrators (Gyrozen, Inchon, Korea) and stored at −20 °C.

Thirty microliters of lysates collected before and after immunoaffinity purification were used for western blot analysis to evaluate the success of immunoprecipitation. Primary antibodies against HLA class I A/B/C (# ab1262377, Abcam, Cambridge, UK), GAPDH (#ab181602, Abcam), and β2M (# ab75853, Abcam) were used. Detection was performed using horseradish peroxidase-labeled goat anti-rabbit IgG (H + L) (# A0208, Beyotime) and Pierce™ ECL western blotting Substrate (Thermo Fisher Scientific, Waltham, MA, USA).

### 2.3. LC-MS/MS Analysis of HLA-I Peptides

Dried HLA-I peptides were re-dissolved in buffer D [2% ACN in 0.1% formic acid (FA)] and then analyzed using an Orbitrap Fusion™ Lumos™ Tribrid™ mass spectrometer (Thermo Fisher Scientific, Waltham, MA, USA) coupled to UltimMate 3000 HPLC (Thermo Fisher Scientific, Waltham, MA, USA) as previously described [25]. In brief, the peptides were separated in an in-house packed column (150 μm × 35 cm, 1.8 μm particle size) with a 95-min gradient at a flow rate of 0.5 μL/min using 5−25% buffer E (98% ACN in 0.1% FA) for 75 min, 25−35% buffer E for 3 min, 35−80% buffer E for 8 min, 80% buffer E for 7 min, and finally a gradient reduced concentration of buffer E from 80% to 5% for 2 min. MS/MS data were acquired in the data-dependent acquisition (DDA) mode. The main parameters of the mass spectrometer were: spray voltage 2 kV, positive mode, 350–1500 *m*/*z* scan range, 60,000 mass resolution, and auto gain control (AGC) value of 1.0e5 within 50 ms maximum injection time for MS1, dynamic exclusion duration of 30 s, intensity threshold of 2 × 10^4^, number of dependent scans for MS2 at Top 30, isolation window of 1.6 *m*/*z*, HCD normalized collision-energy of 30%, 15,000 mass resolution and AGC value of 2 × 10^4^ within 50 ms maximum injection time for MS2, include charge states with 2–6.

### 2.4. Immunopeptide Validation Using Parallel Reaction Monitoring Assay

Synthetic peptides were generated by the Genscript Biotech Corporation (Jiangsu, China). The native and synthetic peptides were analyzed on an Orbitrap Fusion™ Lumos™ Tribrid™ mass spectrometer (Thermo Fisher Scientific, Waltham, MA, USA) in the parallel reaction monitoring (PRM) mode to verify the existence of the peptides indicated by the DDA data. The samples were separated using a 180-min gradient at a flow rate of 0.5 μL/min using 5−25% buffer E for 130 min, 25−35% buffer E for 20 min, 35−80% buffer E for 10 min, 80% buffer E for 15 min, and finally 80−5% buffer E for 5 min. The selected peptides were loaded into Skyline [26] (version 21.2, Department of Genome Sciences, University of Washington, Seattle, WA, USA) to create a list of peptide characteristics, including peptide sequences, *m*/*z*, charges, and HCD collision energy. This list was included in the PRM identification. Most of the analysis parameters including spray voltage, scan range, polarity, maximum injection time, and MS1 resolution followed those used in the DDA method. However, MS2 resolution was set to 30,000 and the AGC target values for MS1 and MS2 were set as 4.0e5 and 5.0e4, respectively. PRM raw data were analyzed using Skyline and PDV [27] software (version 1.7.4, Lester and Sue Smith Breast Center, Baylor College of Medicine, Houston, TX, USA).

### 2.5. Calling Mutations from Whole Exome Sequencing (WES) Data

WES data for the colorectal cell line HCT116 were downloaded from the NCBI database (accession number SRR4032411). WES data for the breast cell line HCC1143 was collected from the Cancer Cell Line Encyclopedia (CCLE) (NCBI accession number SRR8619154) [28]. Somatic mutations were detected following GATK’s best practice [29]. Briefly, raw paired-end reads were assessed using fastp (version 0.20.1, HaploX Biotechnology, Shenzhen, China) [30], and reads with adapter contamination or quality scores less than 20 were removed. Filtered clean reads were aligned to the GRCh38 human reference genome using BWA (v0.7.17, Wellcome Trust Sanger Institute, Cambridge, UK) [31]. Mapped reads were sorted using Samtools (version 1.11, Wellcome Sanger Institute, Hinxton, UK) [32], followed by two main post-alignment processing steps: removal of duplicates and base quality score recalibration (BQSR) using the GATK package (version 4.0.9.0, Broad Institute of MIT and Harvard, Cambridge, MA, USA). Finally, variants were detected using Mutect2 (Broad Institute of MIT and Harvard, Cambridge, MA, USA) [33], with SNPs defined by the 1 K genomes project [34] as a panel of normal and filtered through dbSNP [35], and COSMIC [36] database as germline resources, with “G/T” and “C/T” as artifact modes.

### 2.6. Construction of Individualized Protein Database

A customized database was constructed for the LC-MS/MS data search using in-house Perl scripts. Based on mutation-calling results from the WES data, the 24–45 bases upstream and downstream of the mutation site were retrieved. For amino acid sequence translation, if the mutation site was located within the exon, the translation start site was determined on the basis of the reference gene annotation for 1-frame translation. Otherwise, 3-frame or 6-frame translations were performed based on the strand direction (positive strand/negative strand) of the gene itself (intron case), or of the genes surrounding the mutation site (intergenic case). Translated peptides present in the UniProtKB database were eliminated, and affinity prediction analysis of the remaining peptides was performed using NetMHCpan (version 4.1, Department of Bio and Health Informatics, Lyngby, Denmark) [37] to obtain a compact and efficient database. Peptides with a rank of ≤2% were combined with UniProtKB Swiss-Prot proteomes to generate the personalized database.

### 2.7. Analysis of LC-MS/MS Data

MS/MS raw data were searched against personalized custom databases using MaxQuant (version 2.0.3.0, Max-Planck Institute for Biochemistry, Berlin, Germany) [38] and pFind3 (version 3.1.5, Key Laboratory of Intelligent Information Processing of the Chinese Academy of Sciences, Beijing, China) [39]. The tolerances of the precursor and fragment ions were set at 10 ppm and 0.05 Da, respectively. The contaminant database was included, oxidation (M) and deamination (NQ) were selected as variable modifications, and carbamidomethylation (C) was set as a fixed modification. False discovery rate at the peptide level was set at 1%. The digestion mode was set to unspecific. Only peptides with lengths of 8–15 amino acids were retained. Alternatively, pNovo3 was used for de novo peptide identification [40]. This deduces peptide candidates directly from MS/MS data without any reference database. pNovo3 can identify various novel peptides that are difficult to add to the database for searching, such as undiscovered mutations, unexpected modifications, and spliced peptides. Most of the parameters used for the pNovo3 (version 3.1.3, Key Lab of Intelligent Information Processing of Chinese Academy of Sciences, Beijing, China) were the same as those used for the database search, apart from “Open Search”, which was set to “true.”

### 2.8. Retention Time Prediction

Retention times of all identified peptides were predicted using the default parameters in AutoRT (version 1.0, Lester and Sue Smith Breast Center, Baylor College of Medicine, Houston, TX, USA) [41]. The models used to predict retention time were first trained with high-quality peptides identified using MaxQuant (Andromeda score > 100) for each batch of data. The average retention time (RT) was computed for peptides with multiple matched spectra. The training parameters were as follows: the number of epochs was set to 100 (-e 100); the batch size for training was set to 64 (-b 64), enabling a reduced learning rate when a metric stopped improving (-rlr); the number of epochs with no improvement after which training was stopped was set to 20 (-n 20); and the scaling method for RT transformation was set to “min_max” (-sm min_max).

### 2.9. Affinity Prediction and Clustering of the Immunopeptidome

The binding affinity of immunopeptides was predicted using NetMHCpan (version 4.1), with binding affinity (BA) prediction enabled. Peptides with a rank ≤ 2% were considered binders, and peptides with a rank ≤ 0.5% were considered strong binders. For peptides binding to more than one HLA allotype, only the best-ranked HLA allotype, with its corresponding affinity in nM, was reported. Peptide sequences were clustered using the default parameters in GibbsCluster (version 2.0, Universidad Nacional de San Martín, San Martín, Argentina) [42] and motifs were visualized using Seq2Logo (version 2.1, Technical University of Denmark, Lyngby, Denmark) [43].

### 2.10. Analysis of Non-Canonical Peptide Traceability

The human genome sequence (Assembly: GCF_000001405.39) was retrieved from the NCBI database, and RNA-seq reads of the HCT116 cell line were downloaded from the NCBISequence Read Archive (SRA, accession number SRR4228299). Both genomic and transcriptomic k-mer analyses were performed using Jellyfish (version 2.3.0, University of Maryland, College Park, MD 20742, USA) [44], with the k-mer length varying from 24 to 45 and every three bases being a gradient. K-mer peptide datasets were constructed by translating all unique k-mer nucleotide sequences. Translated peptide sequences containing stop codons were excluded, and non-canonical peptides identified by the de novo method were aligned to the k-mer peptide dataset using an in-house Perl script. Peptides that matched the k-mer peptide dataset were considered to have evidence of origin. Small open reading frame (sORF)-encoded peptides were extracted from the SmProt database [45]. An in-house Perl script was written to identify non-canonical peptides in the sORF database.

### 2.11. Gene Functional Annotation for Canonical and Cis-Spliced Peptide Candidates

The “Leading razor protein” from MaxQuant results were extracted as parental protein of canonical peptides. For cis-spliced peptide candidates, proteins with the least sub-fragment intervening length were extracted as parental proteins. The Gene Ontology (GO) enrichment analysis at the “biological process” level was performed using clusterProfiler (version 4.2.1, Southern Medical University, Guangzhou, China) [46] and visualized using enrichplot (version 1.14.1, Southern Medical University, Guangzhou, China) in R (version 4.1.1).

## 3. Results

### 3.1. An Integrated Proteogenomic Approach Identifies the Immunopeptidome in a Colorectal Cancer Cell Line

**Figure 1:**
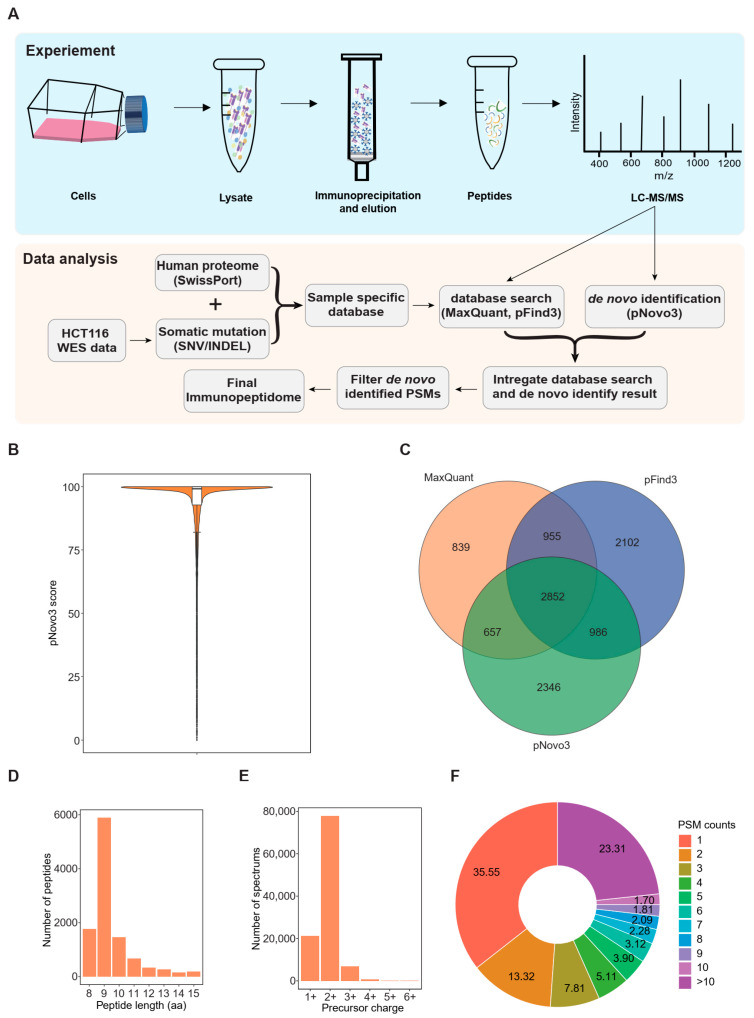
Proteogenomic-based identification of the human leukocyte antigen (HLA) immunopeptidome: (**A**) Schematic representation of experimental and bioinformatic workflow for HLA-I-presented peptide identification; (**B**) Distribution of peptide-spectrum match (PSM) scores reported by pNovo3 for peptides identified by all three items of software; (**C**) Venn diagrams showing the reproducibility of HLA-I-presented peptides identified using the three items of software; (**D**) Length distribution of HLA-I-presented peptides; (**E**) Precursor charge distribution of the HCT116 immunopeptidome; and (**F**) Peptide-spectrum match (PSM) count distribution of the HCT116 immunopeptidome.

We developed a personalized proteogenomic pipeline to acquire the comprehensive immunopeptidome (Figure 1A). We first identified a total of 42,014 somatic variants using WES data from the HCT116 cell line (Figure S1A,B). Since tumor neo-epitopes can originate from various genomic regions, we analyzed somatic variants by preserving all the WES data containing the exon regions and other regions such as intronic and intergenic regions [47]. Then we devised a personalized database construction process with an affinity prediction strategy by adding somatic mutation (include SNV and frameshift INDEL) information to the database to eliminate peptides that are unlikely to be presented by HLA molecules. This finally resulted in 227,143 peptides with mutations (Figure S1C,D). Next, we combined the mutation-bearing peptides with the Swiss-Prot human reference proteome, including isoforms. We then searched 11 batches of LC-MS/MS raw data against the individualized database using two search engines, MaxQuant and pFind3, and obtained 5303 and 6895 peptides at a 1% peptide-level false discovery rate (FDR), respectively. To compensate for the limitation of the database search, we integrated pNovo3 into our pipeline as a de novo identification method to discover atypical peptides not included in the reference database (Figure 1A). To obtain reliable results, we included peptides identified by both pNovo3 and the database search engines in a high confidence dataset, and performed a statistical analysis on the distribution of PSM scores reported by pNovo3 to determine the scoring threshold. The 25th percentile of the PSM score distribution, corresponding to a score of 92.75925, was chosen as a cutoff to filter the pNovo3 results (Figure 1B), leading to the identification of 6841 peptides, 2346 of which were not reported by either database search engines. Overall, the integrative proteogenomic approach applied in this study identified 10,737 unique HLA-I binding peptides, 2852 of which were reported by all three items of software and can be considered high-quality results. Additionally, 839, 2102, and 2346 peptides were exclusively identified by MaxQuant, pFind3, and pNovo3, respectively (Figure 1C).

When investigating the immunopeptidome features, we observed that more than 90% of the peptides were 8–11 mers, with the majority being nonamers, consistent with the length distribution of typical HLA class I peptides. Moreover, in terms of the spectra corresponding to these peptides, the charge state of the precursor ion was predominantly 2+, accounting for up to 72.8% of the spectrum (Figure 1D,E). We further explored peptide-spectrum match (PSM) and found that approximately 64% of the peptides were detected multiple times (multiple PSM) (Figure 1F).

To verify the scalability of our developed proteogenomic strategy, we applied it to a breast cancer cell line HCC1143. LC-MS/MS data from three experiment replicates of the HCC1143 cell line were collected from a previous study [48] and analyzed. As we expected, our pipeline could identify 38.86% more peptides than traditional database search methods. The typical features of HLA-I presenting peptides of HCC1143 cell line were also observed by fundamental characteristic analysis (Figure S2).

### 3.2. Retention Time and HLA-Binding Affinity Predictions Confirmed the Accuracy of the Identified Immunopeptidome 

**Figure 2:**
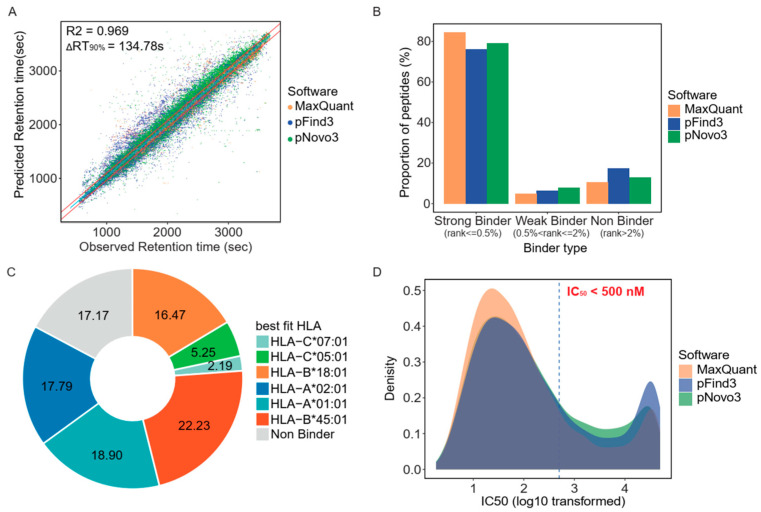
Common features of human leukocyte antigen (HLA)-I-presented peptides of the HC116 cell line: (**A**) Comparison of measured versus predicted retention times of HLA-I-presented peptides showed high correlation (Pearson R^2^ = 0.969). Solid red lines mark the difference between measured and predicted retention times encompassing 90% of all peptides. Solid blue line indicates the fitted linear regression line; (**B**) Proportion of peptides identified by the three items of software under different binder levels. (%Rank ≤ 0.5% for Strong Binder, %Rank > 0.5% and ≤ 2% for Weak Binder, %Rank > 2% for Non Binder)”; (**C**) Distribution of the proportions of peptides with predicted affinity for specific HLA alleles; and (**D**) Distribution of the dissociation constant (IC_50_) predicted by NetMHCpan version 4.1 (Department of Bio and Health Informatics, Lyngby, Denmark).

To evaluate the validity of the immunopeptidome identified using our strategy, we conducted an in silico quality assessment. First, we explored the consistency between the measured and predicted retention times using AutoRT, a deep learning algorithm [41]. To eliminate the interference of batch effects on prediction accuracy, we trained batch-specific models for each batch of data and only peptides with high-quality PSMs (Andromeda score > 100 reported by MaxQuant) were included in the training datasets. After completing the retention time prediction, we analyzed the correlation between the measured and predicted retention times by fitting linear models. The results showed that the predicted retention times were well correlated with the measured values (Pearson R^2^ = 0.969), with 90% of the observations (the difference between the measured and predicted retention times, ΔRT90%) being within 135 s (Figure 2A). We then estimated the binding affinity of the identified peptides using NetMHCpan (version 4.1) and observed that a considerable number of peptides identified by both pNovo3 and database search engines were theoretically HLA-binding peptides with a rank of <2% (Figure 2B). More than 82% of the peptides showed theoretical binding affinity for at least one of the six HLA alleles of HCT116, and most were predicted as binders of HLA-A and HLA-B alleles. HLA-B*45:01 was the most abundant binding allele, with approximately 22.23% of the identified peptides showing a binding affinity for this allele (Figure 2C). Additionally, approximately 69% of the peptides had IC_50_ binding affinities of less than 500 nM, indicating that a considerable portion of the identified peptides had excellent predicted binding affinity (Figure 2D). Overall, the predicted retention time was significantly correlated with the measured values, and a substantial fraction of the identified peptides were theoretical HLA binders. This finding suggests that the in silico quality assessment employed in this study confirmed the accuracy of the identified immunopeptidome.

### 3.3. De Novo Identification Enlarges the Immunopeptidome Landscape of Cancer Cell Lines 

**Figure 3:**
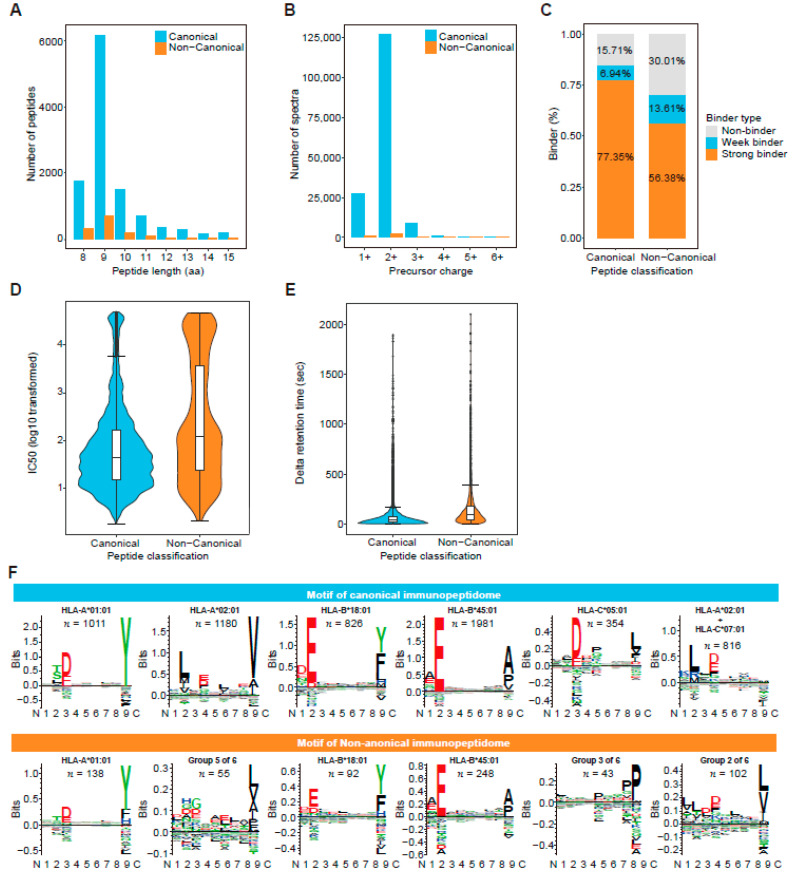
Comparison of features between canonical and non-canonical human leukocyte antigen (HLA) class I immunopeptidomes: (**A**) Comparison of the distributions of canonical and non-canonical peptide lengths; (**B**) Comparison of precursor charge distributions of canonical and non-canonical peptides; (**C**) Comparison of the percentages of peptides bound to HLA class I molecule predicted by NetMHCpan 4.1; (**D**) Comparison of predicted IC_50_ (nM) distributions for canonical and non-canonical peptides; (**E**) Comparison of the distributions of retention time differences between predicted and experimental spectra of canonical and non-canonical peptides; and (**F**) Comparison of six binding motifs in canonical and non-canonical peptides.

Database search engines are commonly used to identify HLA-presented peptides from LC-MS/MS data, and their accuracy and sensitivity rely heavily on the comprehensiveness, integrity, and specificity of an individualized database that cannot be used to identify novel peptides not included in the database. Additionally, existing search engines are not designed for HLA peptides and may be biased toward tryptic peptides. As an alternative approach, pNovo3, a deep-learning de novo sequencing algorithm that overcomes the insufficiency of database search engines, was used to identify novel non-canonical peptides, leading to the discovery of 1293 novel non-canonical HLA-I-presenting peptides. These non-canonical peptides were highly similar to their canonical counterparts in terms of: basic metrics reflecting HLA-presenting peptide features; the length distributions of both types of peptides being concentrated in 9 amino residues; and the precursor charges being both mostly 2+ (Figure 3A,B). Non-canonical peptides also had binder percentage, IC_50_, and retention time differences similar to canonical peptides, although the metric values were slightly lower than those of canonical peptides (Figure 3C–E). Finally, we performed an alignment and clustering analysis on both canonical and non-canonical 9-mer peptides using GibbsCluster (version 2.0) [42], and identified six motifs in both types of immunopeptidomes. The six types of motifs in the canonical peptides were highly consistent with the expected peptide motifs of the HLA-allele genotype of the HCT116 cell line [48] (Figure 3F). However, non-canonical peptides only showed three clear expected motifs, and the other two motifs (Groups 2 and 5) appeared to be mixtures of multiple HLA-I allele-presenting peptides. This may be due to a smaller number of peptides in these two groups, which does not provide enough information for Gibbscluster to unambiguously separate them. In addition, cluster analysis showed that peptides in group 3 ended in proline, a novel motif that was not consistent with any of the six HLA-I allele-presenting peptide motifs in the HCT116 cell line. This might indicate that the de novo sequencing strategy identified a novel set of HLA-presented peptides that could not be identified through a database search.

### 3.4. Traceability Analysis Shows That Some Non-Canonical Peptides Result from Different Omics-Level Variations

**Figure 4:**
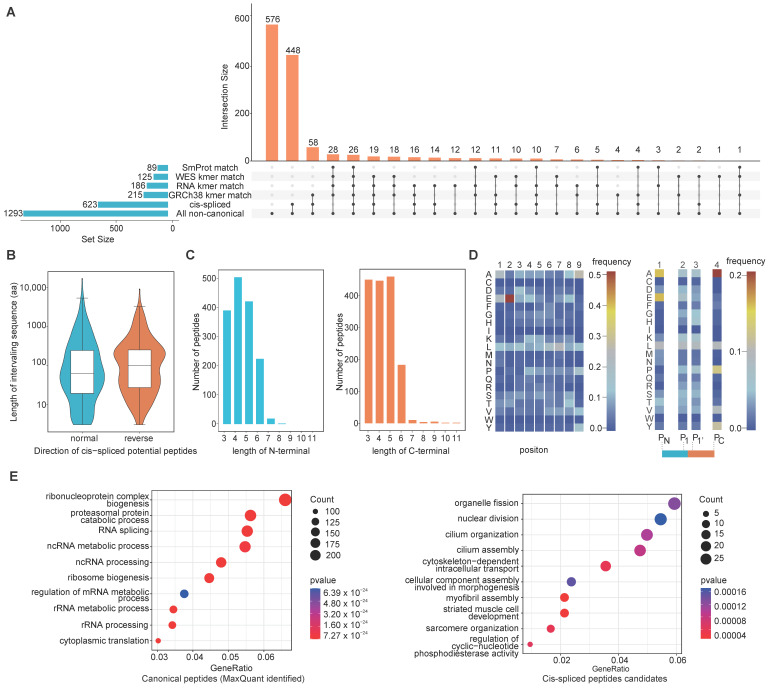
Features of non-canonical peptides identified using de novo identification: (**A**) Upset diagram illustrates the intersection of the non-canonical peptides and the matrix layout for all interaction patterns of the non-canonical peptides at various omics levels, ordered by size. The black circles in the matrix indicate the sets that are part of the intersection; (**B**) Distribution of the lengths of sequences between two spliced fragments (i.e., the intervening sequence) of normal and reverse cis-spliced peptides; (**C**) Distribution of the lengths of N- and C-terminal spliced fragments of cis-spliced peptides; (**D**) Heatmap showing frequencies of the amino acids at each residue of the 9-mer cis-spliced peptide (left) and amino acid preferences at N- terminal, C-terminal, and the boundary of the potential splice site of cis-spliced peptides (right); and (**E**) Top 10 enriched GO terms at the “biological process” level for the parent proteins of canonical peptides identified by MaxQuant (left) and cis-spliced peptides candidates (right), respectively.

We performed traceability analysis at various omics levels to understand the possible origins of non-canonical peptides. We used Jellyfish [44] to conduct k-mer analysis at the genomic, exomic and transcriptomic levels using the human reference genome sequence (GRCh38), HCT116 cell line WES data and HCT116 cell line RNA-seq data, respectively. We first assigned the k-mer 24–45 bases in length, with a gradient at every three bases, as the non-canonical peptides ranged mainly from 8 to 15 amino acid residues. We then translated unique k-mer nucleotide sequences into amino acid sequences, excluding those containing stop codons, and constructed an amino acid k-mer peptide dataset. Finally, we matched the non-canonical peptides back to the k-mer peptide datasets to explore the possible origins of the non-canonical peptides. We found 215, 125 and 186 non-canonical peptides in the k-mer peptide dataset generated from genomic and transcriptomic level data, respectively, with 91 peptides being present at all omics levels (Figure 4A). A total of 269 unique non-canonical peptides were shown to have genomic, exonic or transcriptomic origins. 

For 215 non-canonical peptides with origin evidence at the genomic level, we mapped the corresponding nucleotide sequences of those peptides back to human genome sequences under the version of GRCh38.p13. We define non-canonical peptides as “Unique-Tag” if all nucleotide sequences of the peptide are located in the same type of genomic region, otherwise as “Multi-Tag”. The results showed that 82.33% (177) of 215 non-canonical peptides could be classified as “Unique-Tag”, although multiple hits were found when mapping their corresponding nucleotide sequences to reference genome sequences. More than half of the “Unique-Tag” peptides (89) could be classified as being of the non-exonic origin. The “Multi-Tag” peptides were also mainly intronic (30) and intergenic (31) (Figure S3). Overall, up to 70% (152 of 215 non-canonical peptides) are potentially derived from regions other than exons. This may indicate that the non-coding region is an important source of tumor immunopeptidome. Because sORFs have been studied as alternative sources of tumor neoantigens [6,25], we searched for sORF-derived peptides in our non-canonical peptide datasets. We matched the non-canonical peptides with the sORF database containing 393,285 sORF sequences collected from the SmProt database [45]. We found that 89 non-canonical peptides were present in the sORF database (Figure 4A). 

Most peptides bound to HLA molecules are mainly produced by the proteasome, and include linear peptides and some recombined non-contiguous fragments such as spliced peptides [49]. Studies have shown that proteasome-catalyzed spliced peptides can be presented on the cell surface by HLA-I complexes and are immunogenic [50]. Because the origins of approximately 79% of the non-canonical peptides could not be determined using the traceability analysis, we analyzed the possible origin of proteasomal splicing. We divided each non-canonical peptide into two sub-fragments using the k-mer strategy. The minimum k-mer was set to three amino acid residues. The k-mer sub-fragments were aligned to the UniProtKB Swiss-Prot database. Peptides with both sub-fragments aligned to the same protein were considered possible cis-spliced peptides. The protein with the least sub-fragment intervening length was considered to be the final source protein. A total of 623 non-canonical peptides were identified, which represents potential supporting evidence of cis splicing at the protein level (Figure 4A). 

We additionally explored the characteristics of the cis-spliced peptides. Based on Liepe’s study [51], we defined the cis-spliced peptide with the two sub-fragments oriented from the N- to C-terminus of the parental protein as a normal cis-spliced peptide, and the cis-spliced peptide oriented in reverse order as a reverse cis-spliced peptide. We observed similar numbers of normal and reverse cis-spliced peptides, with 316 and 307 cis-spliced peptides in the normal and reverse orientations, respectively. The distribution of the lengths of the sequences between the two spliced fragments (termed the intervening sequence) in both normal and reverse cis-spliced peptides was mainly centered around 100 amino acid residues (Figure 4B). The length of the intervening sequence was four-fold longer than that used by Liepe et al. in their spliced peptide study using colon and breast carcinoma cell lines [52]. We also observed similar distributions of N- and C-terminal sub-fragment lengths of the cis-spliced peptides, ranging from 3 to 5 amino acid residues (Figure 4C). Because the second and ninth residues usually serve as anchor positions for HLA-presented peptides, we analyzed the amino acid frequency to determine whether a preference for specific amino acids at the anchor site also existed in the cis-spliced peptides. The results showed that glutamate was over-represented at the second residue position. Alanine and leucine were also highly represented at the ninth and seventh residue positions, respectively. In addition, we computed amino acid distributions at P_N_, P_1_, P_1′_, and P_C_ positions of the cis-spliced peptides. We observed no clear preference for specific amino acids at the cleavage sites of the cis-spliced peptides, implying that the choice of splice sites during proteasome-catalyzed peptide splicing is random (Figure 4D). We performed GO annotation for the gene sets of the cis-spliced peptides and the canonical peptides. Notably, the GO features for the gene sets of these two groups are different. Specifically, the source genes of canonical peptides are mainly involved in RNA processing-related processes (Figure 4E), while the source genes of cis-spliced peptides are mainly involved in cell cycle-related processes (Figure 4F). These results suggest that the formation of cis-spliced peptides may be limited to some specific genes, and that these genes are closely related to cell dividing.

### 3.5. Proteogenomics Integrating HCT116 Cell Line-Specific SNV/INDEL Mutation Information Identifies 8 Mutation-Bearing Neo-Epitopes

**Figure 5:**
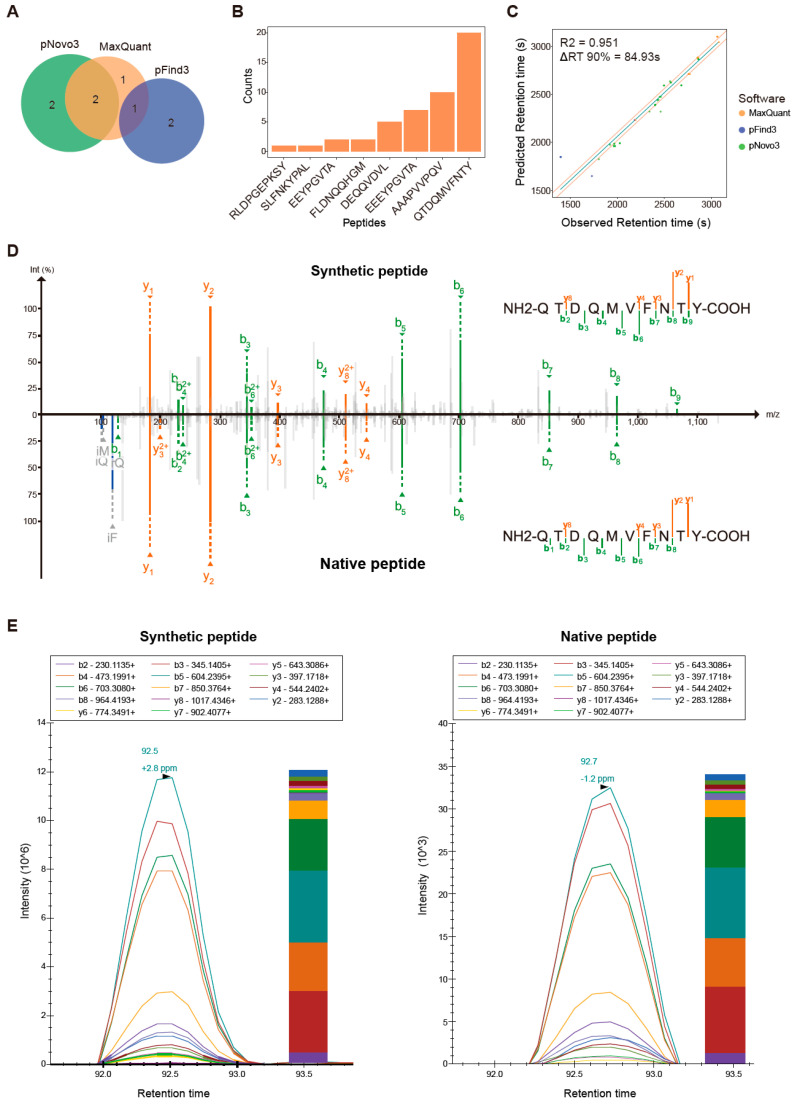
Features of tumor-specific neo-epitopes identified using an integrated proteogenomic approach: (**A**) Venn diagrams showing the overlap in tumor-specific mutant peptides identified by three items of software; (**B**) Distribution of peptide-spectrum match (PSM) counts of eight tumor-specific mutant peptides; (**C**) Comparison of measured versus predicted retention times of eight mutant peptides; (**D**) Mirror diagram comparing the experimentally obtained mutant peptide spectrum (bottom) with its corresponding synthetic peptide spectrum (top) for mutant peptide QTDQMVFNTY; (**E**) Parallel reaction monitoring verifies the consistency of mutant peptides (right panel) with their corresponding synthetic equivalents (left panel) for mutant peptide QTDQMVFNTY. The intensity graph displays parallel reaction monitoring (PRM) peak signals of fragment ions. The bar graph shows the normalized peak areas of all fragment ions for the peptide. The contribution from each fragment ion is shown in a different color in the bar graph.

As somatic mutation is one of the essential sources of tumor-specific neo-epitopes, we used LC-MS/MS data to identify neo-epitope candidates harboring tumor-specific mutations. We first detected somatic mutations in the HCT116 cell line by analyzing whole-exome data, and then integrated the mutation-bearing peptides into the UniProtKB Swiss-Prot database to build an individualized reference database. We used MaxQuant and pFind3 as database search engines to analyze the LC-MS/MS data. Four and three mutant peptides were identified by MaxQuant and pFind3, respectively, using a stringent FDR of 0.01 at the peptide level. Additionally, we used the pNovo3 software as a complementary de novo peptide identification method and discovered six additional mutant peptides. A total of eight mutation-bearing neo-epitopes were identified, one of which was identified by both MaxQuant and pFind3, and two of which were identified by both MaxQuant and pNovo3. One, two, and two mutant peptides were identified specifically by MaxQuant, pFind3, and pNovo3, respectively (Figure 5A). This indicates that using a combination of analytical methods can increase the number of mutant peptides identified. Six of the eight mutant peptides were supported by multiple spectra (>2 spectra, Figure 5B). The mutated peptide, QTDQMVFNTY, had up to 20 supporting spectra. The predicted retention time of each mutant peptide was highly correlated with the experimental retention time, with a Pearson’s correlation coefficient (R^2^) of 0.951. The predicted retention times for 90% of the mutant peptides differed from the corresponding experimental retention times by less than 85 s (Figure 5C). All peptides were predicted to be strong binders, with a minimum IC_50_ value of 15.85 nM for the peptide QTDQMVFNTY (Table 1). These data show the validity of neo-epitope identification in this study. Four of the eight mutant peptides identified have been reported in previous studies [25,48,53,54], while the remaining have not been reported in any literature or patents. To experimentally validate the accuracy of the mutant peptide identification, we compared their spectra with those of their synthetic counterparts. The spectra of the synthetic peptides were strongly correlated with the spectra of the experimental data (Figure 5D, Figure S4). We further performed a PRM assay to confirm the validity of mutant peptide identification. Six of the eight mutant peptides had relative peak intensities, fragment ion overlays, and retention times similar to that of their synthetic equivalents (Figure 5E, Figure S5). Overall, our newly developed proteogenomic approach performed well and showed high accuracy in identifying tumor-specific neo-epitopes even after careful in silico inspection and rigorous experimental validation.

## 4. Discussion

In this study, we developed an integrative proteogenomic approach for conducting an exhaustive and in-depth immunopeptidome analysis of colorectal cancer tumor cell lines. The approach combined two database search engines, MaxQuant and pFind3, and the de novo tool, pNovo3. This facilitates rigorous screening and comprehensive analysis of mass spectrometry data. Using this approach, we identified 10,737 epitopes in HCT116 cells, including 1293 non-canonical peptides. In addition, eight neo-epitopes containing somatic mutations were identified, four of which are novel. 

Our integrative approach effectively increases the number of identified immunopeptidomes. The database search strategy based on the elegant intersection of MaxQuant and pFind3 implemented in our study was shown to be effective. Although the number of epitopes identified by these two search engines differed (approximately 5303 and 6895 epitopes identified by MaxQuant and pFind3, respectively), our strategy reasonably compensated for the algorithmic gap, reporting 8391 peptides. Six mutation-bearing neo-epitopes were also found by database search, four in MaxQuant and three in pFind3. In addition, our approach, which combined a de novo pipeline, boosted the epitope pool. Using the de novo pNovo3 tool, we identified 6841 epitopes, including 4 mutation-bearing and 1293 non-canonical neo-epitopes. The scalability of our novel proteogenomic strategy was also proved on the breast cell line HCC1143. Tumor-specific antigens are potential targets for immunotherapy and have clinical applications. Current research on neoantigens has focused on peptides derived from somatic mutations, including SNVs and INDELs. However, other genomic, transcriptomic, and proteomic alterations that could contribute to the cancer neoantigen landscape, including gene fusion [17], alternative splicing [55,56], RNA editing [57], and proteasome-catalyzed peptide splicing [49,50] have not been well understood. We identified 597 non-canonical peptides that were not attributed to somatic mutations and could not be traced to any specific source. Most basic metrics reflecting the characteristics of these non-canonical peptides were comparable to those of canonical peptides, indicating that the identification of non-canonical peptides is accurate and credible. Their binding motifs were also largely consistent with those of canonical peptides, except for those non-canonical peptides containing a novel motif that was absent in canonical peptides, suggesting that de novo strategies can complement the identification of HLA-presented peptides.

Applying the traceability analysis to data generated from different omics methods identified 269 epitopes at various omics levels, accounting for only 21% of the non-canonical peptides. By examining potential non-canonical epitopes derived from proteasome-catalyzed peptide splicing, we found that approximately half of the non-canonical peptides were spliced epitopes. Among them, 448 non-canonical peptides could be attributed only to peptide splicing, with no evidence of other omics-level origins. Although several studies have reported the presence of a considerable number of spliced peptides in the tumor immunopeptidome [49,51,52,58], the proportion of cis-spliced epitopes has not been determined conclusively. Liepe et al. believed that more than 30% of the immunopeptidome consists of cis-spliced peptides [51], while Roman et al. believed that only 2–6% of the peptides are cis-epitopes [59]. Cis-spliced peptides accounted for 4.2% of the total immunopeptidome (34.65% of non-canonical epitopes) in our study, consistent with Roman’s study. In addition, we found that the source genes of the cis-spliced peptides are functionally related, specifically enriched in cell cycle-related pathways. This functional enrichment may be useful for explaining the mechanism of cis-spliced peptide formation, and we will subsequently test it in more samples to confirm its reliability. A considerable proportion of novel non-canonical peptides were identified using our proteogenomic approach, indicating that our understanding of the mechanism underlying the generation of tumor immunopeptidomes is still limited. Even though we used different traceability analyses, there were still 576 non-canonical peptides (45% of all non-classical peptides) the origin of which was not determined, indicating that the analysis of tumor neo-epitopes should consider additional sources of variation. In addition, the immunogenicity of the canonical and non-canonical peptides identified by our integrative strategy needs to be further validated as neo-antigens for their potential application in immunotherapy.

## 5. Conclusions

This study describes a novel approach for LC-MS/MS epitope analysis for proteogenomic integration that can be used to identify additional immune epitope candidates, including somatic mutation peptides and non-classical peptides. This approach provides an effective process that can be applied to epitope analysis of clinical tumor samples.

## Figures and Tables

**Table 1:** List of mutant peptides identified in HCT116 cells.

Sequence	HLA	IC_50_ (nM)	Mutation Locus	Gene	Protein	AA Change	Wild Peptides Identified	Reference
QTDQMVFNTY	HLA-A*01:01	15.85	chr8:23258741	CHMP7	Q8WUX9	p.A324T	Yes	[25,48,53,54]
RLDPGEPKSY	HLA-A*01:01	1193.67	chr9:35750732	RGP1	Q92546	p.S110P	No	[53]
AAAPVVPQV	HLA-A*02:01	175.14	chr6:149796499	PCMT1	P22061	p.A168V	No	[53]
DEQQVDVL	HLA-B*18:01	771.4	chr20:31605574	ID1	P41134	p.N63D	Yes	-
EEEYPGVTA	HLA-B*45:01	106.68	chr22:17726722	BCL2L13	Q9BXK5	p.I216V	Yes	[48]
EEYPGVTA	HLA-B*45:01	448.13	chr22:17726722	BCL2L13	Q9BXK5	p.I216V	No	-
SLFNKYPAL	HLA-A*02:01	11.61	chrX:115643440	PLS3	P13797	p.N372S	No	-
FLDNQQHGM	HLA-C*05:01	20.86	chr1:88804485	PKN2	Q16513	p.R459Q	No	-

HLA, human leukocyte antigen; AA, amino acid.

## Data Availability

The whole-exome sequencing and RNA sequencing data of HCT116 and HCC1143 cell line analyzed in this study are publicly available on the Sequence Read Archive (SRA) database (accession number: SRR4032411, SRR4228299 and SRR8619154). The mass spectrometry data of HCC1143 cell line are publicly available on the PRIDE database with the data set identifier PXD000394. The LC-MS/MS data of HCT116 cell line that support the findings of this study have been deposited into the CNGB Sequence Archive (CNSA: https://db.cngb.org/cnsa/) of CNGBdb with accession number CNP0002990 (accessed on 5 May 2022).

## Supplementary Materials

The following supporting information can be downloaded at: https://www.mdpi.com/article/10.3390/cancers14123016/s1. Figure S1. The landscape of somatic mutation and theoretically mutation-bearing peptides of HCT116 cell line; Figure S2. Overview of the HLA-I immunopeptidome features of the HCC1143 cell line; Figure S3. Genomic region distribution of the non-canonical peptides with potential origin at the human reference genome level; Figure S4. Mirror diagram comparing the experimentally obtained spectrum (bottom) with its corresponding synthetic peptide spectrum (top); Figure S5. Parallel reaction monitoring verifies the consistency of mutant peptides (right panel) with their corresponding synthetic equivalents (left panel).
