# Supplementary material for: A Novel Proteogenomic Integration Strategy Expands the Breadth of Neo-Epitope Sources
Source: Cancers (Basel). 2022 Jun 19;14(12):3016. doi: 10.3390/cancers14123016 (PMC9220843; doi:10.3390/cancers14123016)
Supplement: Supplementary file 1 [file cancers-14-03016-s001.zip › cancers-1739414-supplementary.pdf]

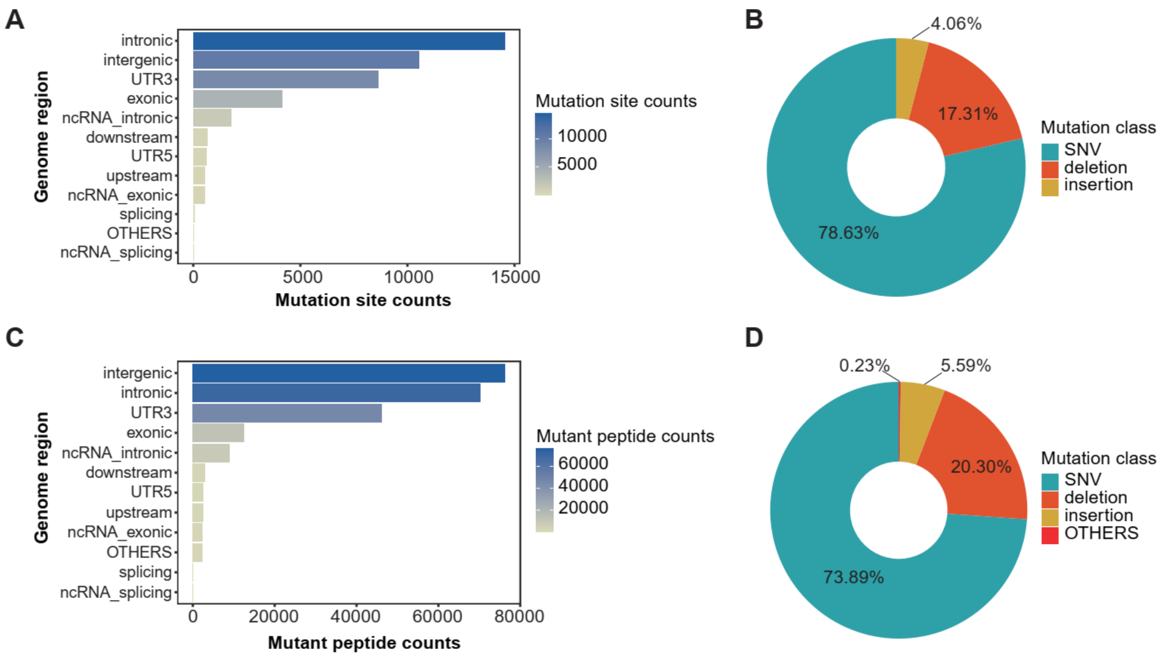

Supplementary Figure S1. The landscape of somatic mutation and theoretically mutation-bearing peptides of HCT116 cell line.

A. Genomic region distribution of somatic mutation sites; **B.** Classification of somatic mutation sites; **C.** Genomic region distribution of theoretically mutation-bearing peptides; **D.** Classification of mutation types contained by theoretically mutation-bearing peptides.

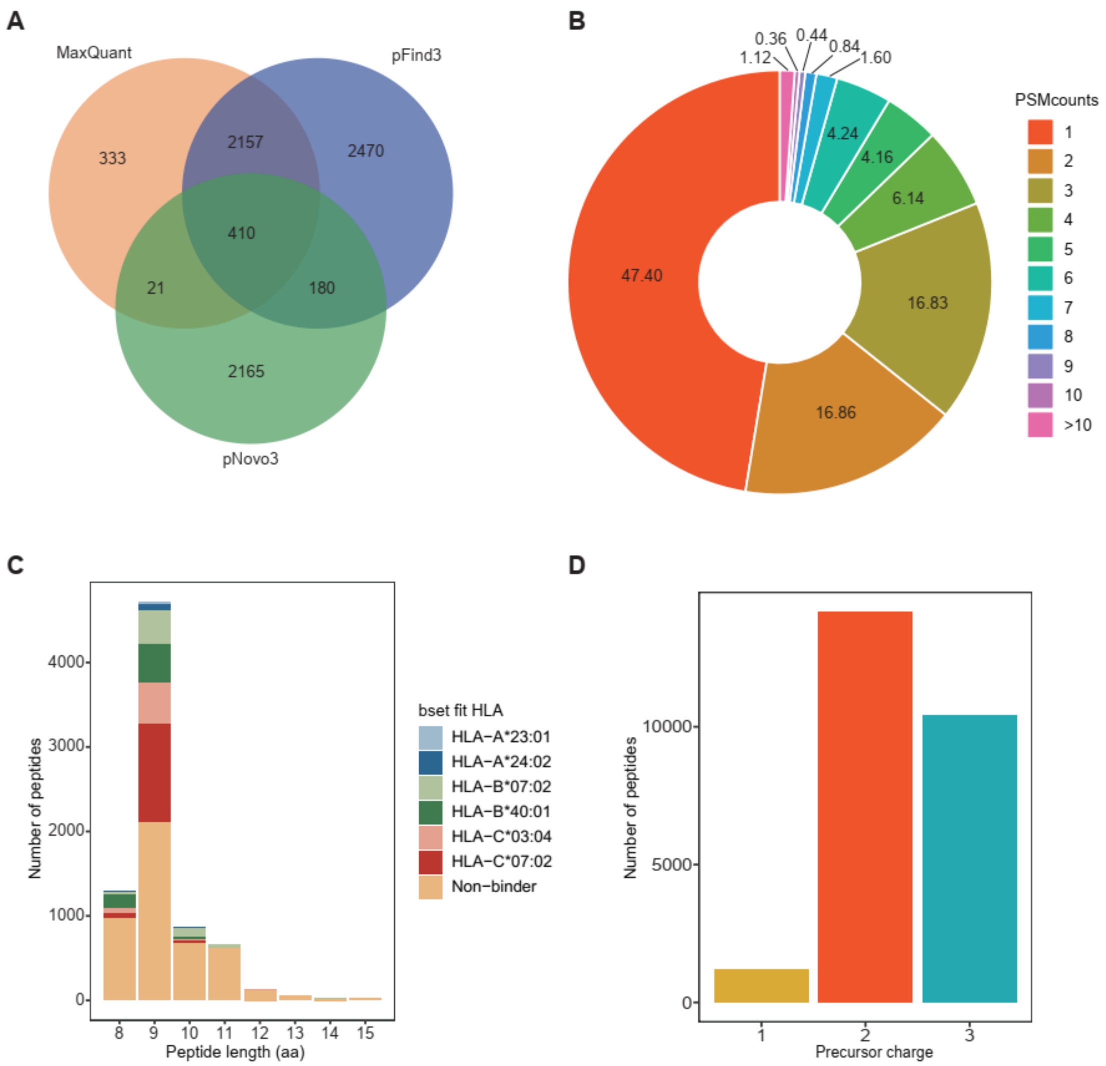

Supplementary Figure S2. Overview of the HLA-I immunopeptidome features of the HCC1143 cell line

A. Venn diagrams showing the reproducibility of HLA-I-presented peptides of the HCC1143 cell line identified using three software; **B.** Peptide-spectrum match (PSM) count distribution of the HCC1143 immunopeptidome; **C.** Length distribution of HLA-I-presented peptides of the HCC1143 cell line. Colors represent the fraction of peptides with predicted binding affinity to a particular HLA allele determined by NetMHCpan 4.1; **D.** Precursor charges distribution of the HCC1143 immunopeptidome.

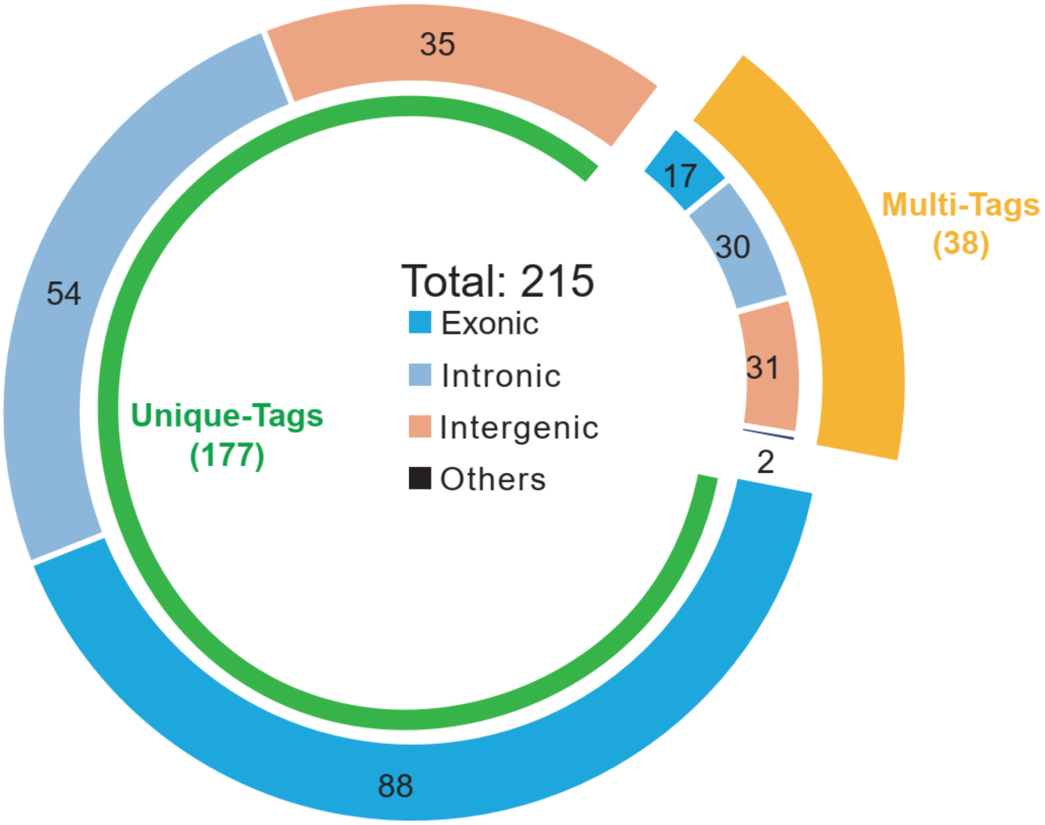

Supplementary Figure S3. Genomic region distribution of the non-canonical peptides with potential origin at the human reference genome level.

All the nucleotide sequences corresponding to 215 non-canonical peptides were mapped back to the GRCh38 human genome sequences. According to their genome coordinates, the mapped sequences were classified and tagged as exonic, intronic, and intergenic, while those that failed to locate were tagged as "Others". A non-canonical peptide is defined as a "Unique-Tag" (green arc) if all nucleotide sequences of the peptide are located in the same type of region. Otherwise, it is a "Multi-Tag" (yellow arc).

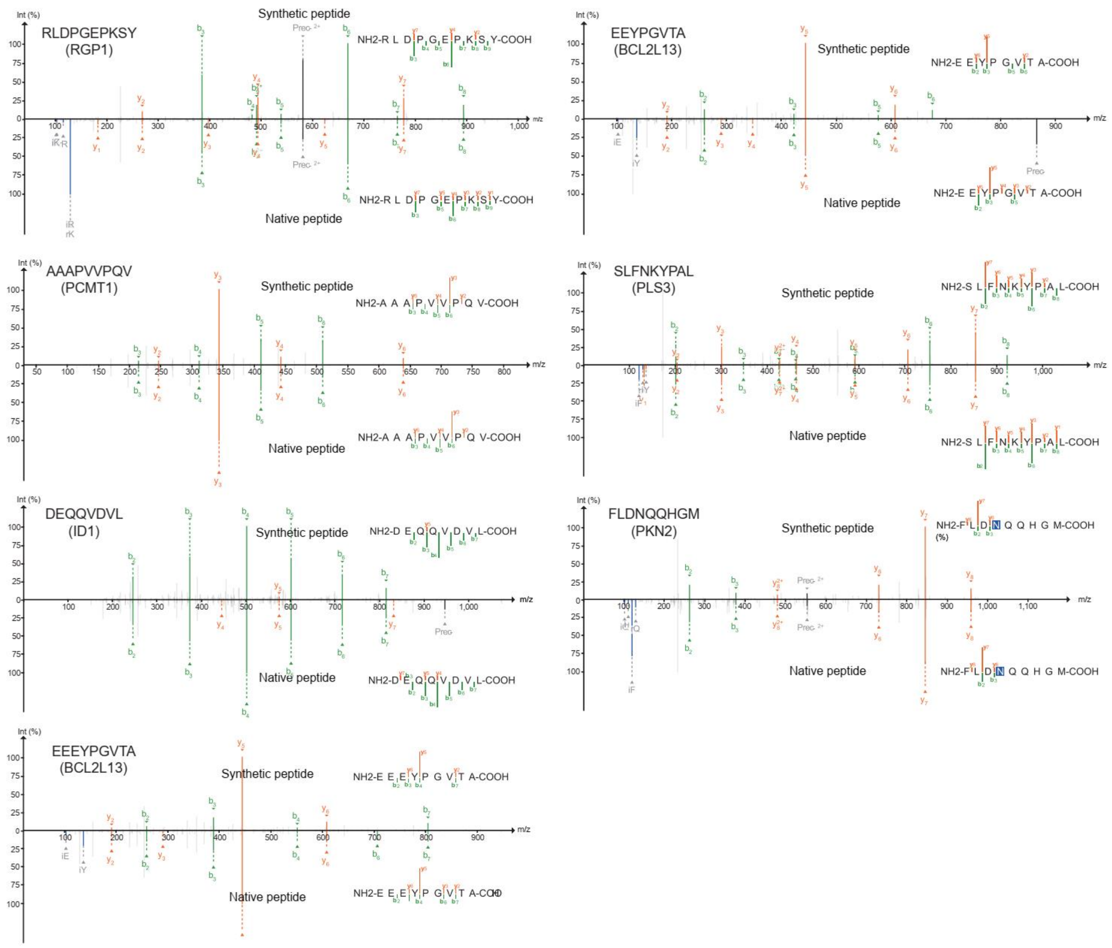

Supplementary Figure S4. Mirror diagram comparing the experimentally obtained spectrum (bottom) with its corresponding synthetic peptide spectrum (top).

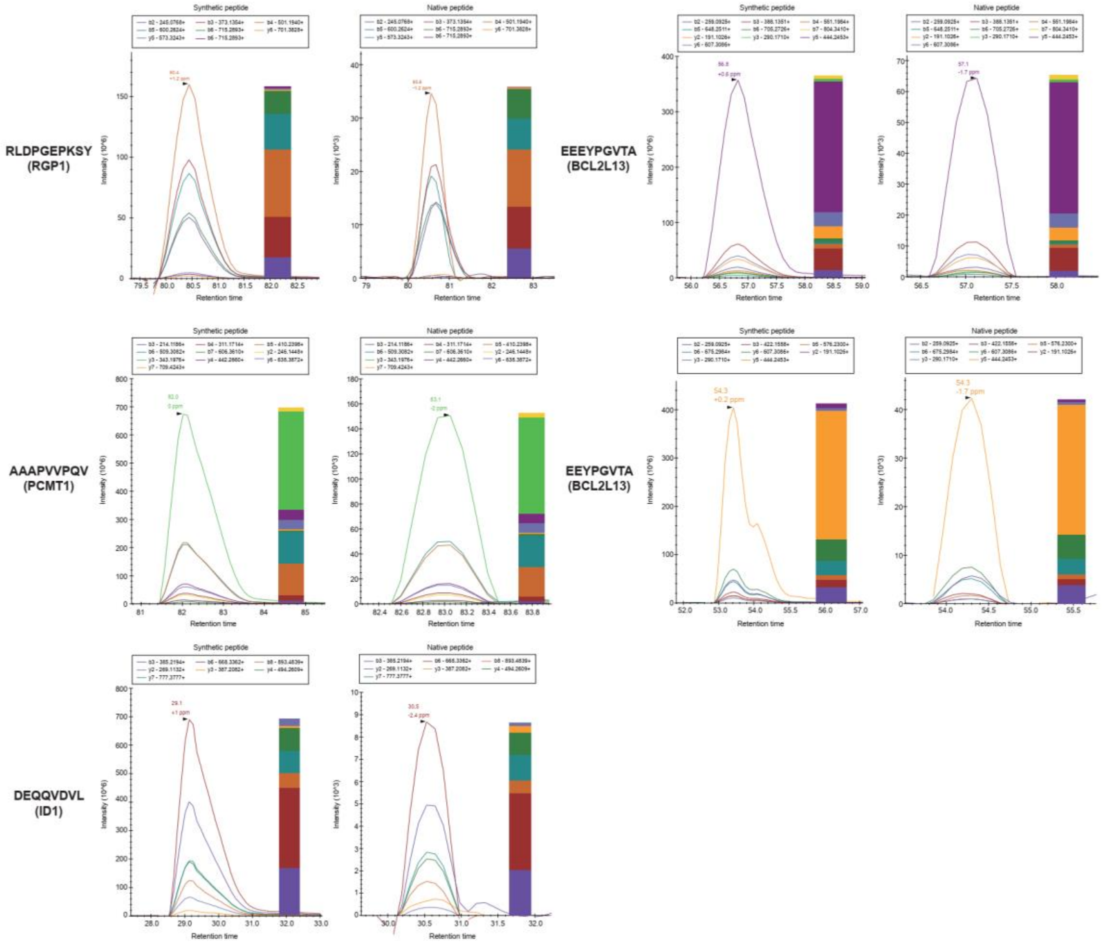

Supplementary Figure S5. Parallel reaction monitoring verifies the consistency of mutant peptides (right panel) with their corresponding synthetic equivalents (left panel).
